# The description and definition of Emergency Department Pharmacist Practitioners in the United Kingdom (the ENDPAPER study)

**DOI:** 10.1007/s11096-019-00799-2

**Published:** 2019-03-16

**Authors:** D. Greenwood, M. P. Tully, S. Martin, D. Steinke

**Affiliations:** Division of Pharmacy and Optometry, University of Manchester, Oxford Road, Manchester, M13 9PT UK

**Keywords:** Emergency care, Emergency department, Examination skills, Pharmacist diagnosis, Pharmacist practitioner, Prescriber, United Kingdom

## Abstract

*Background* Due to a shortage of emergency department doctors and nurses, hospitals have started to employ pharmacists who have additional clinical skills, known as Emergency Department Pharmacist Practitioners, to help deliver services. *Objective* To describe, compare and define the Emergency Department Pharmacist Practitioner role. *Setting* UK emergency departments. *Method* Using a purpose developed questionnaire hosted on a tablet computer, Emergency Department Pharmacist Practitioners were asked to report their contribution to patient care and the wider emergency department over 10 work days. *Main outcome measure* Emergency Department Pharmacist Practitioners’ work. *Results* Twenty Emergency Department Pharmacist Practitioners from 15 UK hospitals were recruited. Of 682 patients cared for, 4.8% (n=33) were of blue triage category (least urgent) and 4.1% (n=28) red (immediate need). Specific activities to inform patient diagnosis included clinical examinations (264/682 patients, 38.7%) and the review of investigation/test/procedure results (270, 39.6%). For treatment, EDPPs prescribed a total of 603 medicines (for administration in the ED) to 266 patients (39.0%) and performed procedures for 63 (9.2%). Eleven of the practitioners also took on the role of designated care provider (i.e. the healthcare professional with overall clinical responsibility) for at least some of their patients. From application of the care typology, all 20 practitioners carried out both ‘traditional’ and ‘practitioner’ activity and 9/20 of them sometimes provided more ‘practitioner’ than ‘traditional’ care to individual patients. Seven key role attributes were identified including how these pharmacists support patients with medical complaints and injuries of any severity and at any stage of their visit. *Conclusion* Emergency Department Pharmacist Practitioners combine traditional clinical pharmacy activities with more hands-on medical practise including being designated care provider. The role is versatile in that care and support provided to patients and the wider emergency department professional team is varied and therefore somewhat adaptable to situations which present.

## Impacts on practice

- There is a role for pharmacists with additional clinical skills training within the emergency department as part of a multidisciplinary team.
- Clinical pharmacists with additional clinical skills training can take on overall clinical responsibility for patients as a practitioner.
- Traditional pharmaceutical care may currently be an unmet patient need in UK emergency departments.

## Introduction

The UK emergency department (ED) typifies the NHS in that care is readily and freely available to anyone. It is a bridge between primary and secondary care where all types of trauma and medical conditions can be treated. In part due to its accessibility, visits to EDs in England increased by 25% between 2007/8 and 2017/8 to 23.9 million [1]. Alongside increased demand, ED performance, as measured by department’s ability to see, treat and either admit or discharge 95% of patients within 4-h, has declined [2]. In March 2018, only 84.6% of patients at EDs in England were managed within 4-h—the worst performance on record [3]. Reduced ED performance has been attributed to a shortage of healthcare professionals to staff departments [4]. Health Education England (HEE) have invested in non-medical professions to supplement these shortages and meet patient demand [5]. Historically the focus has been on nurses but more recently, pharmacists [6].

In 2010, Collignon and colleagues investigated 25 EDs and found that 18 (72%) had some kind of pharmacy service [7]. These were generally limited to indirect care activities i.e. guideline development, but sometimes patient focused i.e. drug history taking. Historically, other countries such as the United States have provided a more involved and patient-facing ED pharmacy service [8]. These often have numerous dedicated and specialist ‘Emergency Medicine Pharmacists’ who reduce medication error rates, reduce the time taken to treat critical patients, and support antimicrobial stewardship.

To develop a workforce of practitioners who could independently manage patients and provide pharmaceutical care in underserved EDs, in 2015, Health Education North West commissioned Manchester Pharmacy School to deliver the ‘Advanced Specialist Training in Emergency Medicine’ (ASTEM) programme [9]. Aimed at clinical pharmacists, the programme trains pharmacists to prescribe independent of medical supervision, and to manage and diagnose patients as a practitioner within a multidisciplinary team led by a consultant doctor. Currently, there is no common recognised training pathway for pharmacists to work in an enhanced clinical role with many other qualifications available e.g. ‘Advanced Clinical Practice’. Provided by many institutions, this is a more general qualification not specific to an emergency care setting or pharmacists, with graduates titled ‘Advanced Clinical Practitioners’ (ACPs) [10]. Graduates of various qualifications now work in an ‘enhanced clinical role’ in UK EDs. A recent qualitative study of pharmacist ACPs’ integration into 3 EDs concluded they are accepted by other professions and “make a positive contribution to workload” [10].

Whilst a 2015 study concluded that pharmacists with additional clinical training *could* manage 36% of ED patients as part of a multidisciplinary team, their *actual* role and contribution to very busy EDs is unknown [11]. Anecdotally, during any given work day, they undertake both traditional clinical pharmacy work (e.g. check the clinical appropriateness of prescriptions) and more novel ‘practitioner’ activities (e.g. perform clinical examinations), for the same and different patients, but this needs to be confirmed.

## Aim of the study

This study aimed to describe, compare and define the role of UK ED pharmacists who had completed additional clinical skills training, collectively termed Emergency Department Pharmacist Practitioners (EDPPs).

## Ethics approval

All procedures performed in studies involving human participants were in accordance with the ethical standards of the institutional and/or national research committee and with the 1964 Helsinki declaration and its later amendments or comparable ethical standards. Approval granted by an NHS ethics committee; reference 17/YH/0275. Informed consent was obtained from all individual participants included in the study.

## Method

A self-administered survey method was used whereby EDPPs recorded their work over 10 workdays.

### Recruitment

Past and current ASTEM students were forwarded a study flyer, as were hospital Chief Pharmacists for onward distribution. Advertisements were published on social media and in professional journals. Those recruited were forwarded a pre-study questionnaire which confirmed eligibility and collected information such as post-graduate education.

### Inclusion and exclusion criteria

Eligible pharmacists worked in any ED and had completed additional clinical skills training. This training could either be a short course i.e. phlebotomy, or a more comprehensive postgraduate certificate i.e. ASTEM. Those with only a post-graduate diploma in hospital pharmacy were ineligible.

### Data collection instrument and tool

EDPPs reported their ED activities over 10 workdays using a two-part questionnaire (named ENDPAPER-Q) on a tablet computer. Part 1 focused on patient care activities and Part 2 on their contribution to the wider ED. On 10 workdays of their choosing, EDPPs completed Part 1 every time they contributed to a patient’s care, and Part 2 twice a day.

Both parts of ENDPAPER-Q were developed according to published best practise [12–15]. First, 3 EDPPs from different hospitals were each observed for 1 day and questions which could capture their activities developed. Following pilot testing with these 3 EDPPs, some questions were re-worded.

Part 1 of ENDPAPER-Q was structured according to the NHS medical note pro-forma, as questions about both traditional and practitioner care could be mapped to this. The Emergency Care Data Set (Version 11), used in the ED to record patient information, further informed the structure and question development [16]. Completion required answers to a minimum of 31 questions (mostly tick-box) and additional fields appeared if certain answers were chosen (a maximum of 978 fields were possible, albeit highly unlikely). Part 2 was a single section of 9 open questions focused on indirect care activity e.g. development or review of ED guidelines, and support provided to other professionals e.g. education and training. Participants were posted a tablet computer with ENDPAPER-Q pre-loaded, and printed instructions about their use. These instructions were supported by 13 brief training videos.

### Development of a Clinical Pharmacy Care Typology

A Clinical Pharmacy Care Typology was produced which defines direct patient care activities as either ‘traditional’ or ‘practitioner’. When applied to data captured by ENDPAPER-Q, the typology supported the description, comparison and definition of EDPP roles. An expert panel of 70 hospital pharmacists and pharmacy researchers were recruited via e-mail or Twitter™ and surveyed as to whether a junior pharmacist working in acute medicine would undertake 85 direct patient care activities (parent categories taken from ENDPAPER-Q Part 1): yes (regularly/rarely) or no (never). A junior hospital pharmacist, who had completed a clinical diploma which is common in the early years of practise, was chosen as a suitable differentiator between traditional and practitioner activities. Those activities which achieved >50% ‘yes’ votes were defined ‘traditional’, and those with >50% ‘no’ votes defined ‘practitioner’.

### Data processing and analysis

Data were exported to Microsoft Excel^®^ for data management and free-text coding. Patient characteristics were described and direct patient care analysed for EDPPs collectively. Using the typology, the ratio of traditional and practitioner activities undertaken for each patient was calculated, with an overall median ratio and range calculated for each EDPP. Finally, the frequency of activities asked in Part 2 were calculated and free-text answers analysed thematically. To define the EDPP role, key attributes were identified through consideration of inclusion criteria and all study findings.

## Results

### Participants

Twenty EDPPs from 15 different NHS hospitals across the UK were recruited who worked a median of 8 half day (4-h) shifts per week (interquartile range [IQR] 5.75–10) over a median of 4.5 days per week (IQR 4–5) in the ED. All worked in the morning and/or afternoon and/or evening, but none worked overnight or on-call. EDPPs had been a registered pharmacist for a median of 9 years (IQR 6.75–16.5) and had a median of 2.5 years of ED experience (IQR 2–5). Overall, participants had completed 18 different types of qualification/course (Table 1).

**Table 1:** Qualifications/courses completed by number of participants

Qualification/course	No. of participants (N=20) (%)
Independent prescribing	16 (80)
Phlebotomy	5 (25)
Cannulation	5 (25)
Clinical skills (comprehensive course i.e. ASTEM)	5 (25)
Clinical skills (extent unknown i.e. whether short or comprehensive course)	4 (20)
Immediate Life Support	4 (20)
Venepuncture	4 (20)
Clinical skills (short course i.e. 2-day residential course)	4 (20)
Ionising Radiation Medical Exposure Regulations training	3 (15)
Intra-venous drug preparation and/or administration	2 (10)
Safeguarding Children Level 3	2 (10)
Clinical reasoning in advanced practise	1 (5)
Advanced Life Support	1 (5)
Basic Life Support	1 (5)
Medicines administration	1 (5)
Infection control	1 (5)
MSc Medical Toxicology	1 (5)
Other e.g. chemotherapy or manual handling training	5 (25)

### Patients

Data were collected for 682 patients, with EDPPs each reporting care they provided to a median of 29 patients (range 10–89). Patient demographics, including triage data and arrival mode, are given in Table 2.

**Table 2:** Patient demographics

Attribute	No. patients (%)
Total number of patients	682 (100.0)
Female	346 (50.7)
Age (years)	
0–5	23 (3.4)
6–12	13 (1.9)
13–18	30 (4.4)
19–25	41 (6.0)
26–30	35 (5.1)
30–39	74 (10.9)
40–49	68 (10.0)
50–59	74 (10.9)
60–69	74 (10.9)
70–79	103 (15.1)
80–89	112 (16.4)
90–99	35 (5.1)
Triage	
Immediate (red)	28 (4.1)
Very urgent (orange)	83 (12.2)
Urgent (yellow)	148 (21.7)
Standard (green)	180 (26.4)
Non-urgent (blue)	33 (4.8)
Unknown	210 (30.8)
Arrival mode	
Road ambulance	325 (47.7)
Self-presented	310 (45.5)
Unknown	24 (3.5)
Other	21 (3.1)
Air ambulance	2 (0.3)

EDPPs most often cared for patients with a medical condition (254/682, 37.2%) or infection (174/682, 23.5%) (see Table 3). Cardiac-related medical complaints were most common (62/254, 24.4%), and specifically an acute coronary syndrome (13/254, 5.1%). For infections, those of the respiratory system were most common (86/174, 49.4%), specifically of the lower respiratory tract (59/174, 33.9%).

**Table 3:** Frequency of diagnosis categories, with most common diagnosis sub-category and diagnosis given

Diagnosis category	Frequency (%)	Most common diagnosis sub-category (n)	Most common diagnosis sub-category (n)
Medical	254 (37.2)	Cardiac (62)	Acute coronary syndrome (13)
Infectious disease	174 (25.5)	Respiratory (86)	Lower respiratory tract infection (59)
Surgical	65 (9.5)	General surgery (24)	Acute pancreatitis (5)
Soft tissue injury/wound	37 (5.4)	Muscle injury (11)	Lower back (5)
Psychiatric	30 (4.4)	Depression (10)	–
Toxicology	23 (3.4)	Paracetamol overdose (8)	–
Fracture/dislocation	20 (2.9)	Closed fracture (17)	Hip—neck of femur (3)
Musculoskeletal	15 (2.2)	Orthopaedics (6)	Sciatica (3)
No abnormality detected	15 (2.2)	–	–
Environmental/social/not applicable	15 (2.2)	Fall (= 5)	–
Trauma	11 (1.6)	Head injury (4)	Contusion of brain (= 1)
Drug/alcohol related	10 (1.5)	Alcohol withdrawal syndrome (= 3)	–
Childhood condition	6 (0.9)	Medical (6)	Croup (= 2)
Obstetrics/gynaecology	5 (0.7)	Gynaecology (3)	Abscess of labia or vulva (= 1)
Foreign body	2 (0.3)	Alimentary tract (1)	–
Total	682 (100.0)		

### Direct patient care

Eleven EDPPs were the ‘designated care provider’ (i.e. the healthcare professional with overall clinical responsibility) for 262 (34.0%) patients . Table 4 presents the care provided by EDPPs prior to diagnosis by an EDPP or another healthcare professional (such as a doctor), and Tables 5 and 6 the care provided afterwards.

**Table 4:** EDPP care provided prior to diagnosis (*diagnosisby an EDPP or another healthcare professional*)

Activity category (number of potential patients)	Number of patients (%/potential patients)	1st most common activity [n (%/potential patients)]	2nd most common activity [n (%/potential patients)]	3rd most common activity [n (%/potential patients)]
History (682)				
Obtained	583 (85.5)	Drug history (575, 84.3)	History of presenting complaint (314, 46.0)	Medical history (308, 45.2)
Reviewed	606 (88.9)	Drug history (489, 71.7)	History of presenting complaint (402, 58.9)	Medical history (338, 49.6)
Vital signs (682)				
Taken	100 (14.7)	Pulse (92,13.5)	Blood pressure (91, 13.3)	Oxygen saturation & temperature (= 90, 13.2)
Findings reviewed	404 (59.2)	Blood pressure (401, 58.8)	Pulse (392, 57.5)	Temperature (384, 56.3)
Clinical examinations (682)				
Performed	264 (38.7)	Respiratory (145, 21.3)	External body (134, 19.6)	Cardiovascular (131, 19.2)
Findings reviewed	323 (47.4)	Respiratory (173, 25.4)	Cardiovascular (152, 22.3)	Abdominal & external body (= 126, 18.5)
Investigations, tests and procedures (682)				
Ordered/requested	147 (21.6)	Full blood count (89, 13.0)	Urea and electrolytes (85, 12.5)	C-reactive protein (77, 11.3)
Results reviewed	270 (39.6)	Urea and electrolytes (220, 32.3)	Full blood count (215, 31.5)	Liver function tests (188, 27.6)
Tests and procedures (682)				
Performed	48 (7.0)	Urinalysis (dipped the patient’s urine) (21, 3.1)	Arterial Blood Gas analysis (10, 1.5)	Venepuncture (9, 1.3)

**Table 5:** EDPP diagnosis, and management planning and treatment provided after patients have been diagnosed (diagnosed *by an EDPP or another healthcare professional)*

Activity category (number of potential patients)	Number of patients (%/potential patients)	1st most common activity [n (%/potential patients)]	2nd most common activity [n (%/potential patients)]	3rd most common activity [n (%/potential patients)]
Diagnosis by EDPP (682)	238 (34.9)	–	–	–
Management planning (682)				
Produced or enacted clinical management plan	162 (23.8)	–	–	–
Produced pharmaceutical care plan	237 (34.8)	–	–	–
Treatment in the ED (682)				
Prescribing medicines	266 (39.0)	Paracetamol (40, 5.9)	Sodium chloride (32, 4.7)	Salbutamol (24, 3.5)
De-prescribing (stopping) medicines	86 (12.6)	Simvastatin (6, 0.9)	Aspirin (5, 0.7)	Ramipril (4, 0.6)
Clinical check/screen/validation of prescription	257 (37.7)	Paracetamol (35, 5.1)	Co-amoxiclav (25, 3.7)	Bisoprolol fumarate (23, 3.4)
Dispensing medicines	54 (7.9)	–	–	–
Accuracy check of dispensed medicines	36 (5.3)	–	–	–
Administration of medicines	46 (6.7)	Intra-venous (34, 5.0)	Other (13, 1.9)	Oral (5, 0.7)
Advice to professional (proactive)	201 (29.5)	Prescribing advice (156, 22.9)	Administration advice (73, 10.7)	De-prescribing advice (39, 5.7)
Advice to professional (reactive)	103 (15.1)	Prescribing advice (57, 8.4%)	Administration advice (38, 5.6)	De-prescribing & monitoring (= 16, 2.3)
Procedures	63 (9.2)	Infusion fluids (37, 5.4)	Supplemental oxygen (31, 4.5)	Positive airways pressure (9, 1.3)^a^

^a^Continuous positive airways pressure/nasal intermittent positive pressure ventilation/bag valve mask continuous airways

**Table 6:** EDPP monitoring, discharge, and admission activities after patients have been diagnosed (*diagnosed by an EDPP or another healthcare professional*)

Activity category (number of potential patients)	Number of patients (%/potential patients)	1st most common activity [n (%/potential patients)]	2nd most common activity [n (%/potential patients)]	3rd most common activity [n (%/potential patients)]
Monitoring treatment in the ED (682)				
Response to medicine	110 (16.1)	–	–	–
Response to procedures	72 (10.6)	–	–	–
Discharge (altogether from ED and hospital) (271)				
Developed discharge plan	58 (21.4)	Medication review (27, 10.0)	Other (25, 9.2)	Outpatient review (8, 3.0)
Enacted discharge plan	40 (14.8)	Medication review (22, 8.1)	Other (21, 7.7)	Outpatient review (2, 0.7)
Prescribed discharge medicines	75 (27.7)	Co-amoxiclav (9, 3.3)	Prednisolone (8, 3.0)	Amoxicillin/trimethoprim (5, 1.8)
Clinical check/screen/validation of discharge prescriptions	43 (15.9)	Amoxicillin (4, 1.5)	Trimethoprim (3, 1.1)	Aspirin (3, 1.1)
Dispensed discharge medicines	26 (9.6)	–	–	–
Accuracy checked discharge medicines	22 (8.1)	–	–	–
Discharge counselling	188 (69.4)	Medicines and condition (114, 42.1)	Condition (56, 20.7)	Medicines (18, 6.6)
Referral	118 (43.5)	General practitioner (84, 31.0)	Outpatient clinic (21, 7.7)	Community pharmacist (20, 7.4)
Admission to hospital (363)				
Transfer of medicines from ED to ward	112 (30.9)	–	–	–
Write first inpatient prescription chart	107 (29.5)	–	–	–

Five categories of activity were particularly novel to these pharmacists, involving hands-on care or making key care decisions and taking associated responsibility.

### To inform diagnosis: clinical examinations, tests and procedures

EDPPs either performed clinical examination(s), and/or reviewed the findings of these, for 344/682 (50.4%) of patients. Including simple general examinations e.g. of general appearance, 16 different types of clinical examination were performed. Whilst general system examinations e.g. respiratory were most common, other more focused examinations were performed including: examinations of the eye (9/682 patients, 1.3%). There were also 11 different tests/procedures performed to inform diagnosis, urinalysis being most common (21/682, 3.1%).

### Diagnosing

**Fig. 1:**
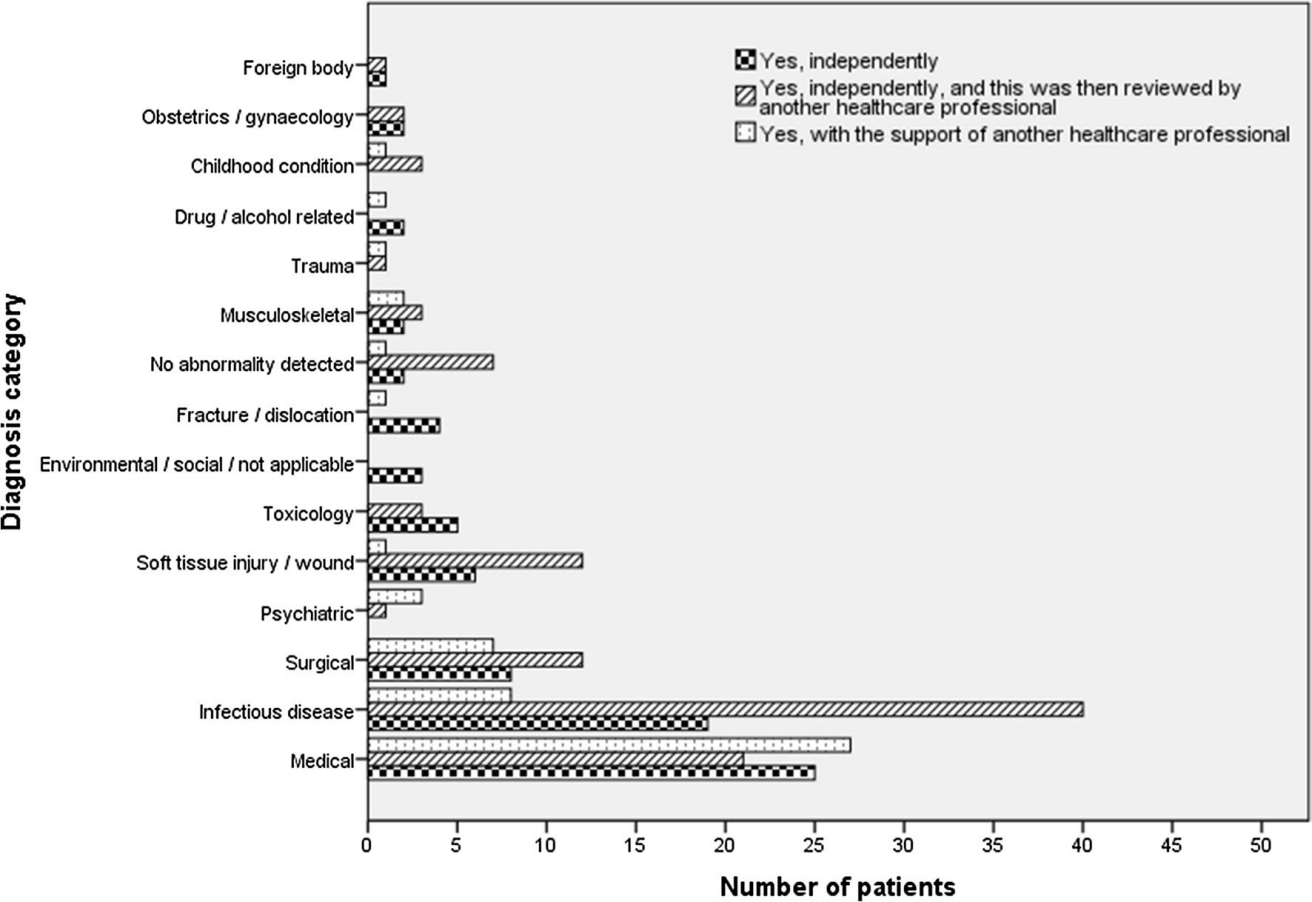
Diagnosis made by EDPPs independently, independently but with review by another healthcare professional, or with the support of another healthcare professional

Of 238/682 patients diagnosed by EDPPs, 79/238 (33.2%) were diagnosed independently, 106 (44.5%) independently (i.e. care provided independently) with review by another healthcare professional, and 53 (22.3%) with the support of another healthcare professional (i.e. care provided collaboratively). Doctors and nurse practitioners, who usually diagnose patients in UK EDs, likely assisted EDPPs with diagnosis. Diagnoses made by EDPPs are presented in Fig. 1.

### Treatment in the ED: prescribing and administering medicines

EDPPs prescribed 1 or more medicines to be administered in the ED for 266/682 patients (39.0%). Details about 603 prescriptions were obtained which consisted of 191 unique drugs. This was a selective sample, as the *detail* of only 3 prescriptions per patient was sought (to minimise data collection workload), prioritising drugs not previously reported for other patients. Aside from those most commonly prescribed (see Table 5), those least prescribed included etanercept (biologic) and digoxin which were both prescribed once. Of 5 possible reasons for prescribing (given for 596/603 prescriptions), re-prescribing of a patient’s regular medicine was most common (291, 48.8%), closely followed by prescribing a new medicine (273, 45.8%). Pharmacists also administered medicines to 46 patients (6.7%), and mostly intravenously (34 patients, 5.0%).

### Treatment in the ED: procedures

EDPPs performed a total of 108 procedures for 63/682 patients (9.2%). Of the 18 different types of procedure performed, some of the less-common yet arguably most clinically involved procedures included: resuscitation/cardiopulmonary resuscitation performed for 7 of 682 patients (1.0%), anaesthetic sedation (3, 0.4%), and modified Valsalva manoeuvre (1, 0.1%). Procedures were performed independently for 56/63 patients (88.9%), with procedures for the remaining 7/63 patients either observed by, or undertaken with the support of, another healthcare professional.

### Discharge: developing and enacting discharge plans

Of 271 patients discharged, 145 (53.5%) had a discharge plan (detailing care required after discharge, if required) developed by ED staff. EDPPs developed at least part of 58 of these (40.0%), most often planning a medication review (27, 46.6%). EDPPs enacted (i.e. put into action) at least part of 40 plans (27.6%). For the 24 plans where further detail was provided, action related to only medication review (22, 55.0%) and outpatient review (2, 5.0%).

### Contribution to the wider ED

Nineteen EDPPs reported their contribution to the wider ED using 148 Part 2 forms, each submitting a median of 10 forms (range 1–12). Six categories of activity were reported with some more common than others: education of individuals/groups (61 forms); risk management (35); guideline development and review (33); ward-round participation (24); financial tasks (20); and other activities (55).

### Typology and its application

From 70 responses from the expert panel, 40 activities were defined ‘traditional’ and 45 ‘practitioner’. Respondents were divided 50:50 about whether ‘prescribing discharge medicines’ was a ‘traditional’ or ‘practitioner’ activity. Given that respondents were asked to consider a junior pharmacist who was not a prescriber, this was defined ‘practitioner’.

### Role comparison

**Fig. 2:**
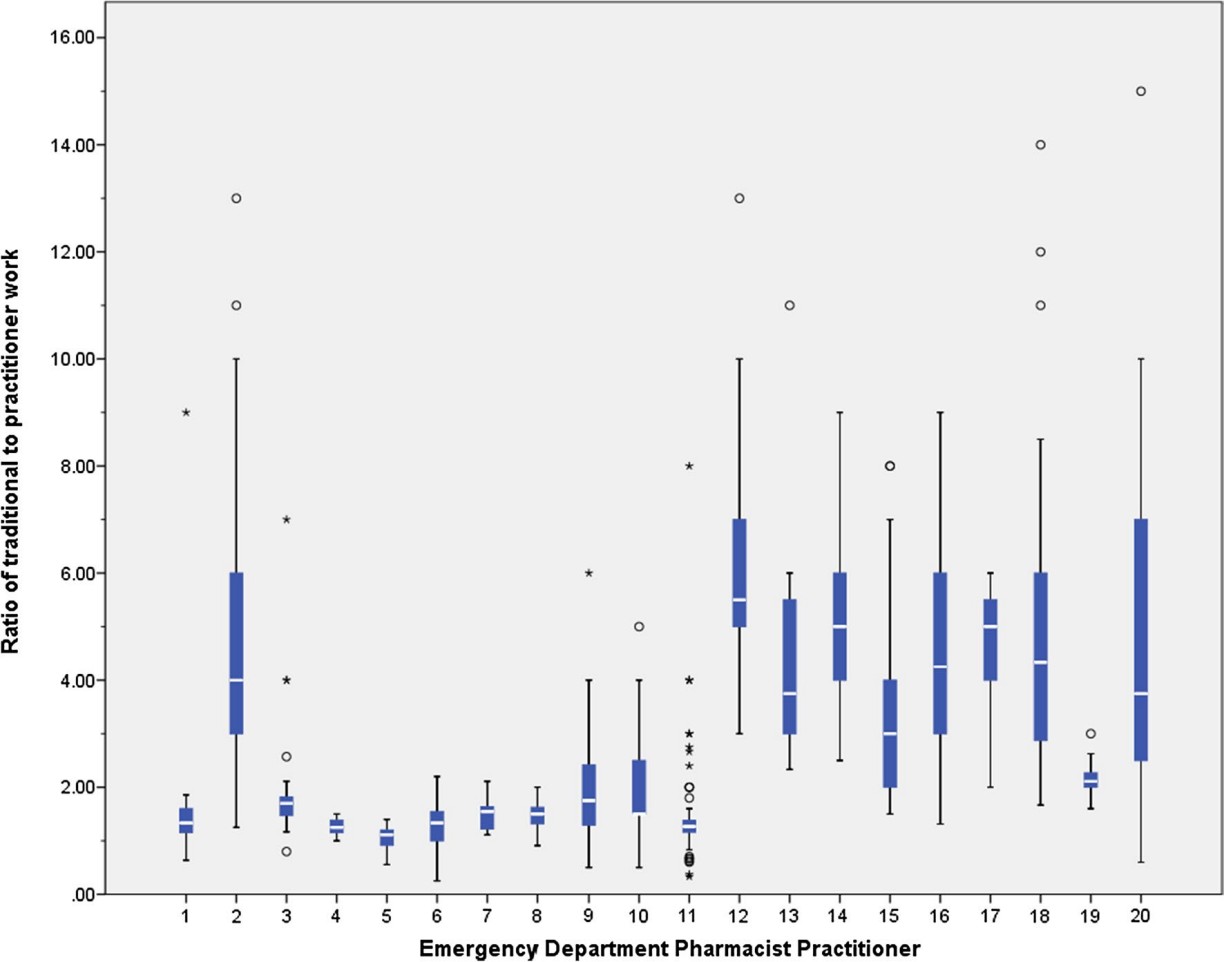
Overall median ratios for each EDPP. The ratio of traditional to practitioner activities, as defined by the newly developed typology, was calculated for each patient cared for. Each box plot represents these ratios for the patients cared for by an EDPP. o = Outliers >1.5 × interquartile range; ★ = Outliers >3 × interquartile range

Overall median ratios of traditional to practitioner work ranged from 1.11 to 5.50. The median ratios for each EDPP are listed in Table 7 and presented graphically in Fig. 2.

**Table 7:** Ratio statistics for each EDPP

EDPP	No. patients	Median ratio	Min. ratio	Max. ratio
1	25	1.33	0.64	9.00
2	89	4.00	1.25	13.00
3	32	1.70	0.80	7.00
4	17	1.25	1.00	1.50
5	42	1.11	0.56	1.40
6	29	1.33	0.25	2.20
7	39	1.55	1.11	2.11
8	11	1.50	0.91	2.00
9	23	1.75	0.50	6.00
10	30	1.50	0.50	5.00
11	87	1.27	0.33	8.00
12	33	5.50	3.00	13.00
13	10	3.75	2.33	11.00
14	20	5.00	2.50	9.00
15	84	3.00	1.50	8.00
16	10	4.25	1.31	9.00
17	12	5.00	2.00	6.00
18	31	4.33	1.67	14.00
19	27	2.11	1.60	3.00
20	29	3.75	0.60	15.00

Whilst all EDPPs provided both traditional and practitioner care, all provided more traditional than practitioner care to more patients. However, 9 EDPPs sometimes provided more practitioner than traditional care to individual patients. With respect to their overall role, EDPPs who provided a greater proportion of practitioner care did so more consistently, whilst those who provided a greater proportion of traditional care did so less consistently.

### Role definition

Seven key role attributes were identified. EDPPs:

- Have completed additional hands-on clinical skills training
- Provide medical and/or pharmaceutical care, and sometimes arrange social care
- Work in any area of the ED
- Support patients with medical complaints and injuries of any severity and at any stage of their visit
- Are sometimes the designated care provider of patients
- Care for patients as part of a multidisciplinary team led by a consultant doctor, and learn from and educate this team
- Undertake indirect patient care activities e.g. develop guidelines

Based on these attributes, EDPPs are:

"Hospital pharmacists who have completed additional clinical skills training and who provide medical and pharmaceutical care to patients with medical complaints or injuries, in any area of the ED and at any stage of their visit. They work as a member of a multidisciplinary team, supporting and being supported by others, and may take overall responsibility as the patient’s designated care provider."

## Discussion

Considering this study in a broader context, various aspects of the EDPP role are particularly novel. EDPPs provide both traditional and practitioner care. This combination supports HEE’s efforts to develop a flexible workforce which can respond to ever-changing patient demand [5]. Through introduction of an EDPP service, those EDs who have limited—if any—pharmacy service could gain 2 types of role from 1 employee; a pharmacist who can both undertake more general pharmaceutical activities but also provide direct patient care when needed. However, with nurses who moved into practitioner roles, this combined approach was thought to compromise their service [17]. Described as the ‘mish-mash’ effect, nurses felt stressed as they were “torn between trying to maintain their new roles, which are comparable to junior doctor level, and being forced to revert to generic nursing roles” [17]. Perhaps, distinct EDPP care pathways or more specific roles could be developed to prevent internal conflict. After their participation in this study, an EDPP chose to revert to a wholly traditional role as they perceived this was most useful to an ED with no other pharmacy input yet many other practitioners e.g. nurse practitioners. To maximise potential impact on patient care, perhaps EDs should first ensure traditional clinical pharmacy services are provided, as other professional groups can become practitioners.

EDPPs were the designated care provider of 232 patients (34.0%). Whilst a small sample size of 682 patients, this proportion is similar to HEE’s suggestion that 36% of ED patients would be suitable for management by a pharmacist [11]. Medicines typical for an emergency care setting were prescribed for 39.0% of patients e.g. analgesics, inhalers, and antibiotics; however, there were some unexpected prescriptions e.g. for the biologic medicine etanercept. An EDPP re-initiated Enbrel^®^ (proprietary etanercept) for a patient who was recently changed to generic etanercept and who visited ED with a suspected allergic reaction to that product. As the prescribing practises of pharmacists change, so should associated legislation and guidance to ensure the correct balance between permitting and limiting autonomy to ensure patient safety.

Of 596 EDPP prescriptions, 273 (45.8%) were for newly initiated medicines. In a comparable study of 1415 pharmacist prescriptions (inpatient setting) by Baqir and colleagues (2014), only 13.0% were for a newly initiated medicine [18]. Whilst this difference could be due to the different clinical settings studied or the different data collection methods, it does suggest pharmacists have the confidence to independently initiate new medicines.

Traditionally the jurisdiction of doctors, EDPPs examined over a third of all patients (38.7%) and carried out many tests and procedures. These examples demonstrate a change in pharmacists towards provision of hands-on patient care using new clinical skills. As with the advent of ED nurse practitioners, hands-on care provided by pharmacists could be a source of conflict with doctors who might feel that their professional boundary is encroached upon [19]. ED staff should be educated on the EDPP role to ensure opinions are informed and thereby reduce misconceptions and allay fears.

A strength of this study is that the proposed role definition is generalisable UK-wide due to broad inclusion of EDPPs with varied training from 15 EDs across the country. Further, the use of a self-administered questionnaire ensured that EDPPs could report their cognitive work which other methods may have failed to capture (i.e. record review). However, a self-administered method was limited in that incorrect information—whether accidental or on purpose—could have been recorded. Another limitation was that EDPPs chose cases to report so could have included only their most ‘impressive’ cases i.e. for patients whom they carried out many activities and interventions. However, other methods e.g. random work-sampling might not have captured less common activities such as diagnosing.

Future research should develop EDPP care pathways which support efficient provision of traditional and practitioner care to the same and different patients. The wellbeing of EDPPs working in these ‘mish-mash’ roles should also be explored and, if necessary, methods to mitigate occupational stress pursued. Any interprofessional conflict, or synergy that could be harnessed, should also be investigated. Finally, to conclude their value, the impact of EDPPs on the quality of care should be evaluated, ideally compared with other practitioners.

## Conclusion

Pharmacists with additional clinical skills can act as designated care provider with overall responsibility for ED patients. Termed EDPPs, they combine traditional pharmaceutical activities with more hands-on clinical practise. The role is versatile in that patient care and support to the ED team is varied and therefore somewhat adaptable to situations which present. For example, EDPPs who work as a designated care provider can fill gaps in doctor and nurse practitioner rotas, something that can only be welcomed given ongoing staff shortages. They can also provide pharmaceutical care that is lacking in some EDs e.g. check prescriptions for clinical appropriateness. Perhaps, EDPPs are the versatile solution to both staff shortages and a lack of pharmacy input.
